# Distinct chemical blends produced by different reproductive castes in the subterranean termite *Reticulitermes flavipes*

**DOI:** 10.1038/s41598-021-83976-6

**Published:** 2021-02-24

**Authors:** Pierre-André Eyer, Jared Salin, Anjel M. Helms, Edward L. Vargo

**Affiliations:** Department of Entomology, 2143 TAMU, Texas A&M University, College Station, TX 77843-2143 USA

**Keywords:** Chemical ecology, Sexual selection, Social evolution, Behavioural ecology

## Abstract

The production of royal pheromones by reproductives (queens and kings) enables social insect colonies to allocate individuals into reproductive and non-reproductive roles. In many termite species, nestmates can develop into neotenics when the primary king or queen dies, which then inhibit the production of additional reproductives. This suggests that primary reproductives and neotenics produce royal pheromones. The cuticular hydrocarbon heneicosane was identified as a royal pheromone in *Reticulitermes flavipes* neotenics. Here, we investigated the presence of this and other cuticular hydrocarbons in primary reproductives and neotenics of this species, and the ontogeny of their production in primary reproductives. Our results revealed that heneicosane was produced by most neotenics, raising the question of whether reproductive status may trigger its production. Neotenics produced six additional cuticular hydrocarbons absent from workers and nymphs. Remarkably, heneicosane and four of these compounds were absent in primary reproductives, and the other two compounds were present in lower quantities. Neotenics therefore have a distinct ‘royal’ blend from primary reproductives, and potentially over-signal their reproductive status. Our results suggest that primary reproductives and neotenics may face different social pressures. Future studies of these pressures should provide a more complete understanding of the mechanisms underlying social regulation in termites.

## Introduction

Social insects are among the most ecologically successful taxa, with colonies containing up to several million individuals. Their success relies on the efficient allocation of these individuals to different colony tasks, such as reproduction carried out by specialized reproductive castes^1^ and other tasks like foraging for food, rearing brood or defending the nest carried out by non-reproducing individuals^2,3^. For social insects, the fitness of non-reproducing workers and reproducing castes is inextricably linked, as workers indirectly increase their fitness by assisting the reproductives^4,5^. For this reason, it is vital that both castes accurately signal and recognize each other to optimize the reproductive output and colony fitness within a given social and environmental context^6,7^ and to efficiently provide each member with the necessary level of resources and care.

Social insect species exhibit diverse communication methods employing visual and chemical signals to communicate reproductive status^7–11^. Chemical communication is the most commonly used modality among social insects to communicate the status of colony members, largely relying on blends of different cuticular hydrocarbons (CHCs) to define a broad range of conditions for each individual, such as their species identity, colony of origin, caste, age, health, reproductive status or fertility quality^12–14^. Compounds unique to female and/or male reproductives, including CHCs, may function as royal pheromones. Royal pheromones often inhibit the development of additional reproductive individuals (i.e.*,* rival queens or kings, or opportunistic cheating workers) to maintain harmony and relatedness within colonies^15^. This may be achieved in different ways by eliciting two types of responses from colony members^3^ through releaser and/or primer pheromones^16,17^. First, royal pheromones can serve as releaser pheromones, inducing a behavioral response in other colony members. Behavioral changes may include eliciting the immediate and continual attention from surrounding workers or inducing policing behaviors. For example, queen pheromones identified in different species of Hymenoptera represent an honest signal from fertile reproductives causing workers to cooperatively refrain from reproduction themselves or to display aggressive policing behaviors that prevent additional reproductives within the colony^18^. Second, royal pheromones can also function as primer pheromones, acting like hormones to impact physiological or developmental processes, including preventing other individuals from developing into reproductives. Queen pheromones in Hymenoptera have been shown to act as ‘queen control’ over reproduction, inducing a physiological change that reduces ovarian activity of nestmates^15,19^. The chemical composition of royal pheromones, as well as the function of various compounds in honest signaling, have been extensively studied in the social Hymenoptera^15,20–22^, including bees^23^, wasps^24,25^ and ants^19,26^, but it relatively little is known about royal pheromones in termites.

Termites developed social life independently from Hymenoptera, but they share many features of their social organization. A major difference stems from the continuous presence of the reproductive male (i.e., king) in termite colonies after their foundation, in addition to the queen. This suggests that termite kings must also express royal recognition pheromones^27^. A second major difference is the presence of another reproductive pathway in many termites, allowing workers or nymphs to develop into neotenics, reproductives that do not develop as fully formed imagoes (primary reproductives). Neotenics can be of two types. Brachypterous neotenics, also called secondary reproductives, arise from nymphs, while apterous neotenics, sometimes referred to as tertiary reproductives, develop from workers^28^. The development of neotenics usually occurs following the loss of the primary king or queen, allowing colonies to continue reproducing even after a primary reproductive has died^29–32^. The development of neotenics in orphaned colonies suggests that queen pheromones have a primer effect which inhibits worker and nymph development into neotenics while the king or queen are still alive^33^. Neotenics effectively assume the same reproductive function as the primary reproductives, and there are often numerous neotenics present in a colony. Their presence also inhibits further neotenic development^34^, limiting the number of reproductives within colonies. This therefore raises the question whether primary and neotenic reproductives produce a common royal pheromone.

Three royal-specific pheromones have recently been identified in termites. In four higher termite species from the subfamily Syntermitinae, primary queens uniquely emit the highly volatile (*E*)-nerolidol, which is absent from kings, workers and soldiers^35^. Heneicosane was recently identified as a royal-specific cuticular hydrocarbon pheromone in *Reticulitermes flavipes*, inducing behavioral responses when presented to workers (i.e.*,* royal recognition releaser pheromone^36^). The presence of this compound was investigated in neotenics, but not imagoes of this species. This compound elicits lateral and longitudinal shaking behaviors by workers and soldiers, which are usually performed in proximity of the queen and king^37,38^. Another royal pheromone was identified in *R. speratus.* It comprises a volatile blend of 2-methyl-1-butanol and n-butyl n-butyrate, which elicits primer effects inhibiting the development of workers into neotenic reproductives^33^ and reducing fecundity of nestmate secondary queens^39^. It remains uncertain whether heneicosane has similar primer effects in *R. flavipes,* or if it only serves as a royal recognition releaser pheromone. In addition, heneicosane was suggested as a royal-specific pheromone based only on the comparison between workers and neotenics from two colonies of *R. flavipes* in North Carolina, USA^36^. Several questions therefore remain unanswered. First, it is unknown whether heneicosane is conserved across all *R. flavipes* populations, or whether distinct populations have evolved different royal recognition pheromones^40^. Second, heneicosane is reported in neotenic individuals, but it is unknown whether this compound is also produced by the primary reproductive castes. Finally, it is not clear whether heneicosane is also a recognition signal for the future reproductive castes (present in nymphs or alates), or only for those who have begun reproducing (present in founding reproductives) or are fully reproductive (present in mature reproductives).

This study aims to further investigate the expression of heneicosane as a royal pheromone by neotenics and primary reproductives of *Reticulitermes flavipes.* First, we assess the presence of heneicosane in neotenics in Texas, USA to test whether this compound is used across geographically distant populations. Second, we further examine royal pheromone composition by investigating additional non-volatile royal-specific compounds produced by both primary reproductives and neotenics. Finally, we measure the expression of heneicosane in primary queens and kings, investigating the ontogeny of its production by measuring this compound at different stages from nymph, alate, newly mated, to reproductively active individuals.

## Results

### Chemical signature of male and female neotenics

**Figure 1:**
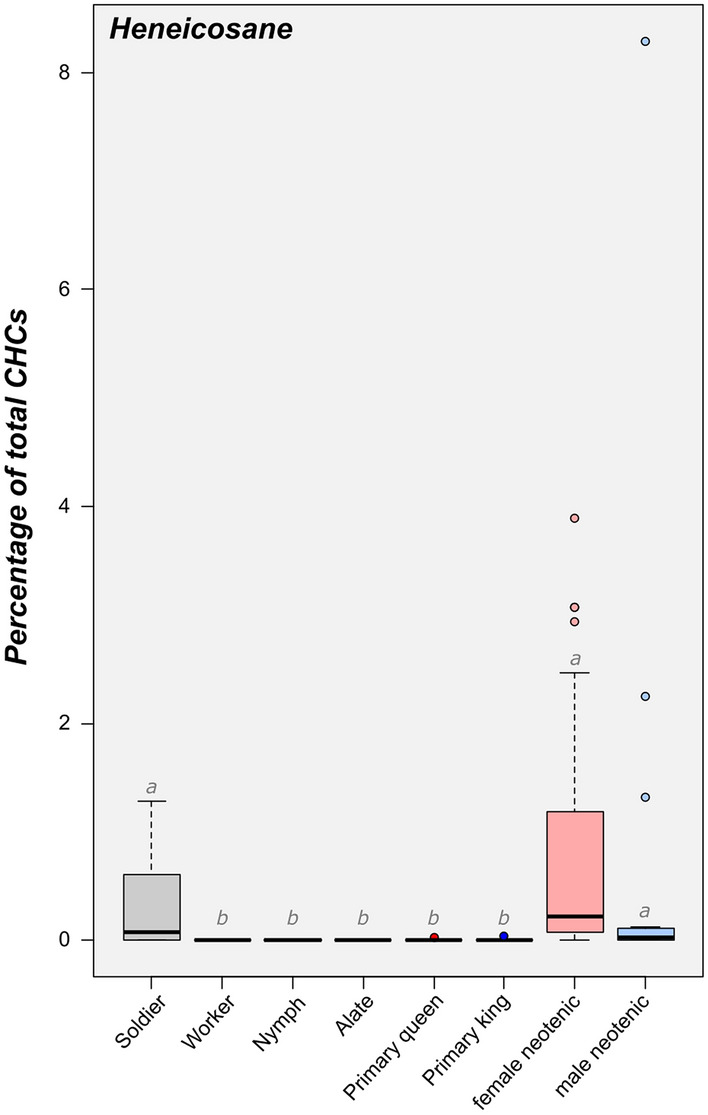
Representation of the relative proportion of heneicosane across castes and sexes of *Reticulitermes flavipes*. Box plots represent median and 1st and 3rd quartile; whiskers include 95% of all observations; individual dots show outlier values. Different lower case letters indicate significant differences between groups. Dark red markers represent primary queens, dark blue represent primary kings, light red markers represent female neotenics and light blue represent male neotenics. Non-reproducing castes (soldier, worker, nymph and alate) are depicted in gray.

The use of heneicosane as a royal pheromone by male and female neotenics was confirmed, since the compound was absent from workers, nymphs and alates, and present in significantly greater quantities in neotenics of both sexes (all *P* < 0.001, after Bonferroni correction for multiple comparisons; Fig. 1 and S1a). Yet, heneicosane was not detected in every neotenic; it was found in 46 out of the 61 females and 8 out of the 15 males. Unfortunately, we did not record whether variation in the production of heneicosane could be explained by the developmental origin of the neotenics (i.e.*,* whether they were nymph-derived or worker-derived). Heneicosane concentrations were not different between male and female neotenics (*P* = 0.889). The level of heneicosane in female neotenics varied with their colony of origin (*P* < 0.001) as well as between female neotenics within a colony; however, it did not vary with the number of female neotenics found within colonies (*P* = 0.918; Figure S2a,b). Interestingly, heneicosane was absent from primary reproductives, but was found in the soldier caste in similar quantities as in male neotenics (*P* = 1.0) and female neotenics (*P* = 0.918, present in seven out of the 10 soldiers analyzed). Notably, individuals (i.e.*,* female and male neotenics, as well as soldiers) that did not produce heneicosane do originate from specific colonies (Figure S2c). The cuticular profiles of *R. flavipes* soldiers comprise many additional compounds not found in other castes (Figure S1b). This includes high quantities of previously identified defensive compounds, such as the terpenes, cadinene and cadinene-aldehyde^41–43^, but also many additional compounds that we did not include in our analyses for the current study. Because we were not focused on identifying soldier-specific compounds, only soldier compounds shared with other castes were included in our analyses. The rich chemical profile of soldiers may explain some overlap with specific royal compounds observed in this study (Figure S1).

**Figure 2:**
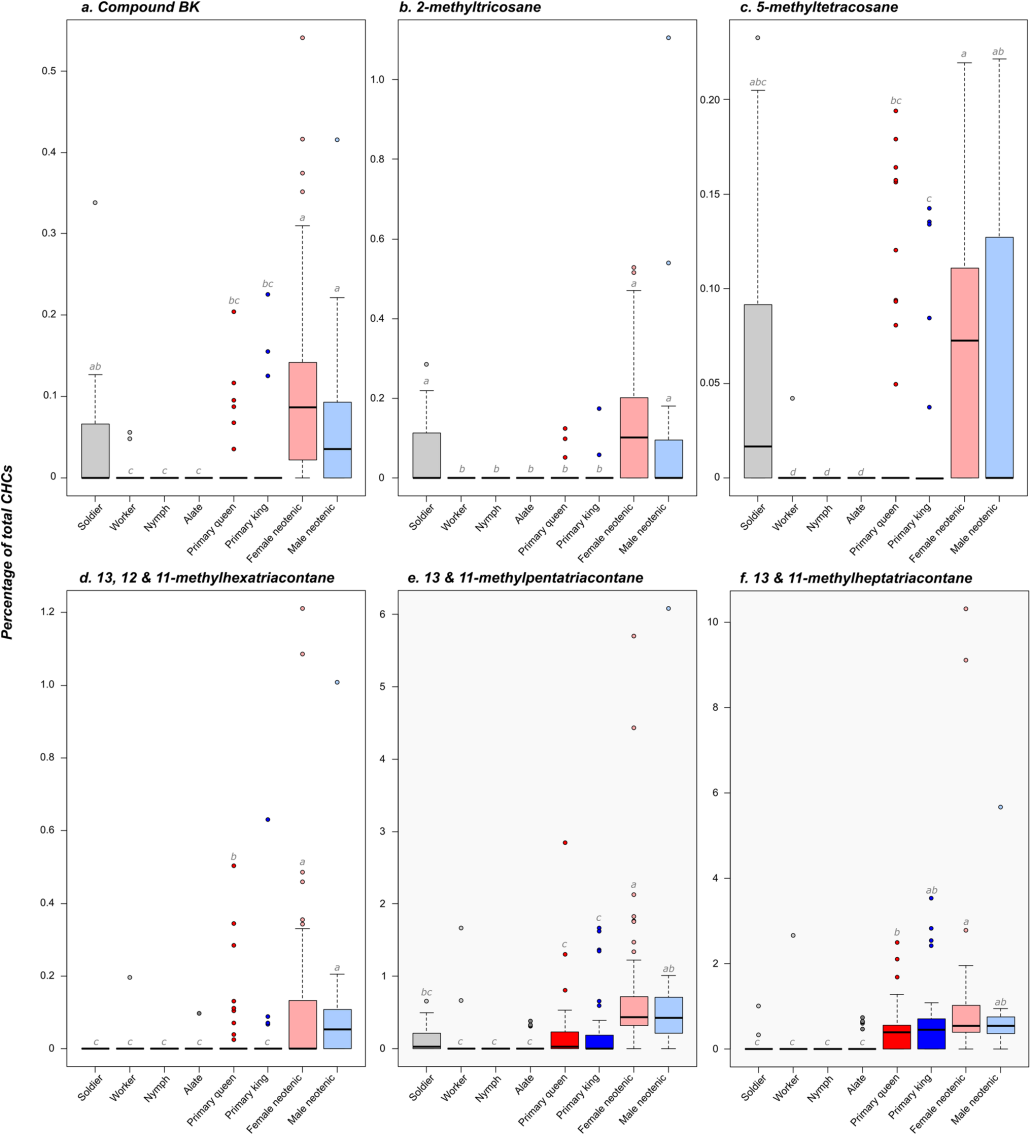
Representation for different castes and sexes of the relative proportion of the six additional compounds potentially used as royal pheromones by the male and female neotenics of *Reticulitermes flavipes* (**a** Compound BK; **b** 2-methyltricosane; **c** 5-methyltetracosane; **d** 13, 12 and 11-methylhexatriacontane; **e** 13/11-methylpentatriacontane; **f** 13, 12 and 11-methylheptatriacontane)*.* Box plots represent median and 1st and 3rd quartile; whiskers include 95% of all observations; individual dots show outlier values. Different lower case letters indicate significant differences between groups. Dark red markers represent primary queens, dark blue represent primary kings, light red markers represent female neotenics and light blue represent male neotenics. Non-reproducing castes (soldier, worker, nymph and alate) are depicted in gray.

During this experiment, we uncovered six additional compounds potentially used as royal pheromones by the male and female neotenics. These chemical compounds are 2-methyltricosane, 5-methyltetracosane, 13- and 11-methylpentatriacontane, 13-, 12- and 11-methylhexatriacontane, 13- and 11-methylheptatriacontane, and the unidentified compound BK (Fig. 2). These compounds were present in higher quantities in neotenics of both sexes than in workers, nymphs and alates, from which they are completely absent or not significantly different from zero (all *P* < 0.01, after Bonferroni correction). Three of these compounds were also produced in higher quantities in male and female neotenics than in soldiers (all *P* < 0.05 for 13- and 11-methylpentatriacontane, 13-, 12- and 11-methylhexatriacontane, and 13- and 11-methylheptatriacontane), but not the three other compounds (all *P* > 0.1 for 2-methyltricosane, 5-methyltetracosane and BK). Like heneicosane, all these compounds were also present in similar quantities in male and female neotenics (all *P* = 1.0). Although these caste differences suggest a role in chemical recognition, we cannot rule out that these heavy chained CHCs have more of a structural function than a role in communication.

**Figure 3:**
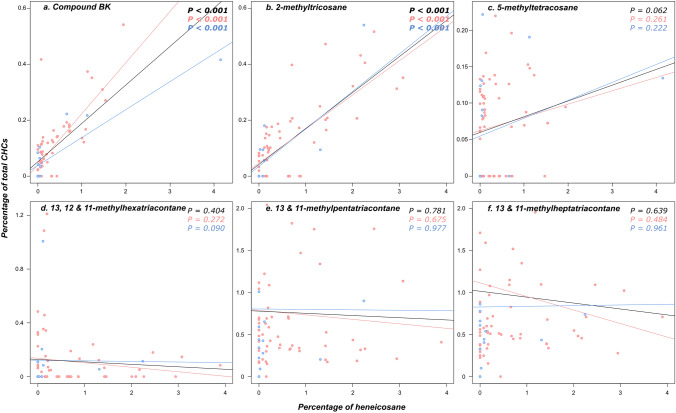
Correlation between the relative proportion of heneicosane and the one of each of the six additional compounds potentially used as royal pheromones by the male and female neotenics (**a** Compound BK; **b** 2-methyltricosane; **c** 5-methyltetracosane; **d** 13, 12 and 11-methylhexatriacontane; **e** 13/11-methylpentatriacontane; **f** 13, 12 and 11-methylheptatriacontane). Female neotenics are marked in red and male neotenics in blue.

Interestingly, these compounds are also present in higher quantities in neotenics than in primary reproductives (all *P* < 0.01). However, these differences are not significant between male neotenics and primary reproductives for 13- and 11-methylheptatriacontane (both *P* = 1.0), or between male neotenics kings and primary queens for 5-methyltetracosane (*P* = 0.352) (Fig. 2). Notably, the concentrations of 5-methyltetracosane and the compound BK correlate with the concentration of heneicosane, meaning that a male or female neotenic producing a low dose of heneicosane also produces a low dose of these compounds (all *P* < 0.001; *P* = 0.062 for 5-methyltetracosane; Fig. 3). One other compound (Tricosane) was produced in higher quantities in neotenics than in other castes (all *P* < 0.001); but we discarded it as a potential ‘neotenic’ royal pheromone since it was also present in substantial quantities in non-reproductive castes (Supporting Information, Figure S2).

### Influence of physiological status on royal pheromone production in primary reproductives

Originally, we planned to investigate the behavioral or physiological factors affecting heneicosane production within primary reproductive individuals over time, measuring this compound at different stages, from alate, mated, to egg-laying primary kings and queens. Therefore, alates from different colonies were sexed and paired to create incipient colonies. CHC profiles were extracted from males and females before pairing, at different periods after mating from two days to 1.5 years (540 days), as well as from two mature primary queens (i.e.*,* fully physogatric) and one primary king collected in the field. Overall, CHC profiles of 116 founding queens and 110 founding kings were analyzed at different stages. As mentioned above, heneicosane was surprisingly absent from primary reproductives, regardless of their age or status and was therefore not different from that produced by the worker caste (*P* = 1.0, for both primary queens and kings). Only the compounds 13- and 11-methylpentatriacontane and 13- and 11-methylheptatriacontane were found in both neotenics and primary reproductives while being absent from the non-reproducing castes. These compounds were present in 87% and 97% of the male and female neotenics, respectively. No chemical compounds were found to be unique to primary reproductives. Four other compounds (pentacosatriene and the unidentified compounds × 15, EZ and FH) were produced in higher quantities in primary reproductives than in the other castes; but were disregarded as ‘primary’ royal pheromones due to their presences in non-reproductive castes (Supporting Information Figure S2).

**Figure 4:**
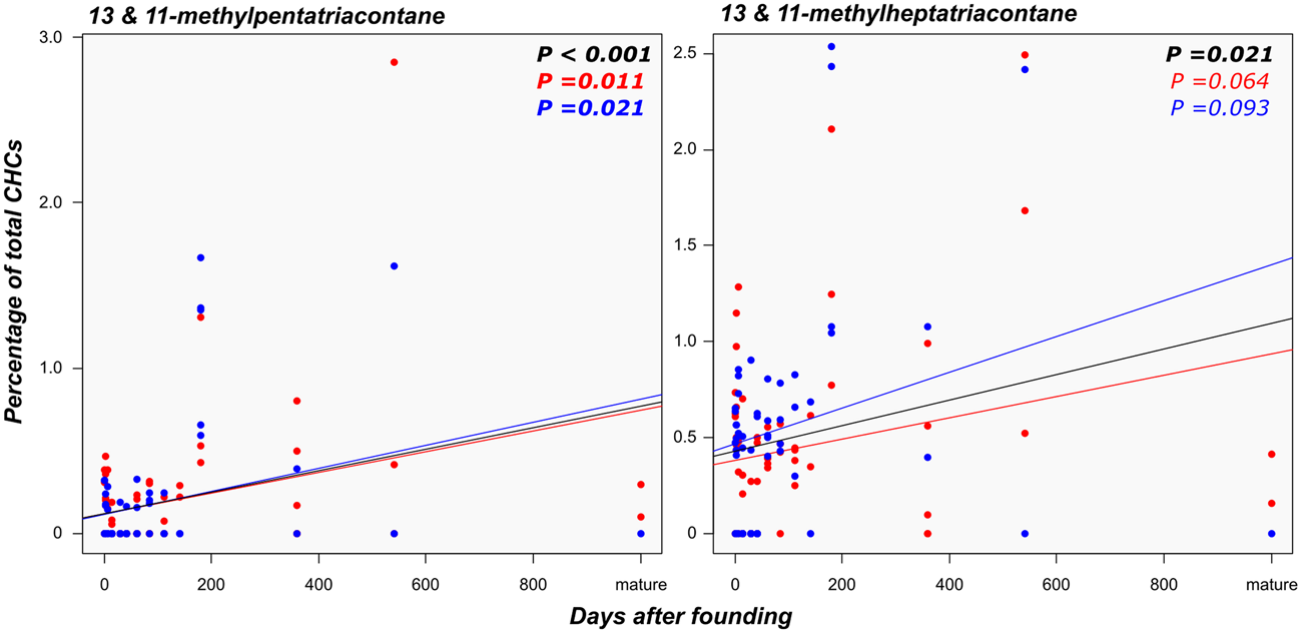
Relationship between the time elapsed since the foundation of incipient colonies and the production of 13/11-methylpentatriacontane and 11/13-methylheptatriacontane in founding primary reproductives.

**Figure 5:**
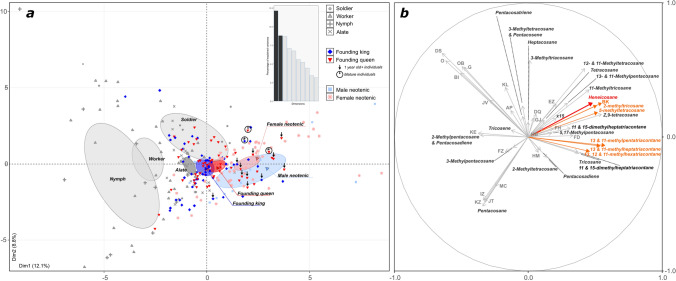
PCA of gas chromatograms from individuals of *R. flavipes* of different castes and sexes. (**a**) Principal component analysis of the 48 GC compounds. Dark red markers represent primary queens, dark blue represent primary kings, light red markers represent female neotenics and light blue represent male neotenics. Non-reproducing castes (soldier, worker, nymph and alate) are depicted in gray. The percent of variance explained by PC1 and PC2 is indicated on the axes. (**b**) PC1 and PC2 eigenvectors are represented for each compound analyzed.

Like the ‘neotenic’ royal compounds, the concentrations of 13- and 11-methylpentatriacontane and 13- and 11-methylheptatriacontane did not differ significantly between primary kings and queens (all *P* = 1.0). Interestingly, the relative abundance of these compounds increased with the age of the individual (Fig. 4). A similar pattern is observed for the primary reproductive-enriched compounds pentacosatriene and the unidentified compound EZ (Supporting Information Figure S3). Overall, individuals clustered according to their castes based on their CHC profiles, with most neotenic reproductives being more similar to primary queens and kings at all the different ages investigated than to non-reproducing castes (Fig. 5).

## Discussion

Our findings provide further insights into the chemical signatures potentially used for caste discrimination in the termite species *R. flavipes*. We first confirmed that heneicosane was more likely to be found in neotenics^36^*,* but that this compound is not consistently produced by all of them, raising the question of additional factors triggering heneicosane production in this caste. In addition, we found that heneicosane was not the only compound specifically produced by neotenics of this species, as they produced six additional chemical compounds absent from workers, three of which were also absent from soldiers. The concentrations of three of these compounds are positively correlated, suggesting that any information these compounds may convey relies upon the strength of the signal rather than on variation in proportions of individual compounds. Remarkably, heneicosane and four of the compounds used by neotenics were not detected in primary reproductives of *R. flavipes,* and the two remaining compounds were produced in higher quantities by neotenics, rather than primary reproductives. In addition, no unique compound was found in primary reproductives. Overall, these results suggest that neotenics of *R. flavipes* evolved a distinct ‘neotenic royal’ chemical blend not found in primary reproductives, and produce higher quantities of the shared reproductive compounds to potentially over-signal their reproductive status.

Reproductive conflicts within colonies occur when several individuals can potentially reproduce. This may happen at least two times during the lifespan of a lower termite colony such as *Reticulitermes*. First, when the primary pair of reproductives establishes their reproductive exclusivity, and second, after the primary reproductives die, at which time potentially every worker and nymph compete to become the next reproductives. This inevitably results in competition over reproduction as all workers and nymphs can develop into neotenics. In our study, we found that neotenics exhibit higher concentrations of the royal compounds that are also found in primary reproductives, and produce a suite of ‘neotenic royal’ compounds, which are not produced by the primary reproductives. These findings suggest that neotenic reproductives may over-signal their fertility or dominance in order to achieve or maintain their reproductive status. Interestingly, the transition from a single pair of primary reproductives to the development of several neotenics is associated with competition among female neotenics and among male neotenics^44^, absent during the first stage of the colony life span. This reduced level of competition for reproduction in the early stage may explain the lower production of royal compounds by primary kings and queens, as their reproductive roles in the colony are more clearly established than those of neotenics. In *R. flavipes*, neotenics compete for breeding positions, which leads to physical aggression initiated by older neotenics and followed by nestmate workers^44^. In addition, sex-specific regulation of reproductive development has been identified, such that the presence of female reproductives inhibits the development of other females reproductives, but stimulates the development of male reproductives and *vice versa*^34^. The mechanism of this inhibition remains unknown but is likely mediated by a chemical signal. In our study, the neotenics may over-signal their fertility to receive more care from the workers, and potentially influence them during reproductive conflicts among neotenics to avoid being killed. Similarly, selection may act on new neotenics to rapidly produce large amounts of royal pheromone to quickly inhibit the development of additional neotenics of the same sex.

This transition from primary reproductives to neotenics may resemble the monogyne and polygyne social forms observed in hymenopteran social insects. In the ant *Lasius niger*, colonies are usually established by several queens, but when the worker force is large enough, workers kill the numerous queens to restore monogyny. In this species, the queen survival rate is positively correlated with queen pheromone production, which is associated with its ovarian development^19^. In monogyne colonies of the ant *Solenopsis invicta,* workers preferentially kill nonphysogatric queens when given a choice between a physogastric and a non physgastric queen in an experimental setting^6^, while in polygyne colonies*,* the most attractive queens receive more food from workers and have a higher oviposition rate^45^. A similar finding is observed in polygynous colonies of *Leptothorax sp.A* and *F. fusca,* in which workers provide better care to the more fertile queen based on CHC signaling^46,47^. Interestingly, queen-queen competition in ants may also be associated with a decrease in queen fecundity through primer and/or releaser effect resulting in their mutual inhibition (*e.g., Linepithema humile*^48^ and *S. invicta*^49,50^). Comparable results have been reported in the termite *R. speratus*, in which queens exposed to queen pheromone exhibited a reduced egg production, despite the primer or releaser role of this pheromone being unknown^39^. It may occur through releaser effects, whereby worker or queen adjust their behavior to provide or beg for a larger amount of food. It may also arise through a primer effect whereby the pheromone acts directly on the queen's neuroendocrine system to reduce egg production^39^.

The transition from a primary queen to female neotenics using similar reproductive signaling compounds may share common features with social parasites, whereby a foreign individual assumes reproductive control by producing reproductive signals similar to the host queen^51^. For example, the parasitic female of the cuckoo bumblebees *Bombus bohemicus* kill and usurp the place of the host queen to take control of worker reproduction in order to ensure her own reproductive success^52^. In this case, the social parasite monopolizes and prevents worker reproduction by mimicking the fertility signal of the host queen^53^. Interestingly, social parasites can also produce additional compounds that the host queen does not. These can be appeasement compounds that reduce host worker aggressiveness, such as in the slave making ant *Polyergus rufescens*^54^, or deterrent and repellent compounds preventing attack, such as in the ants *Rossomyrmex minuchae*^55^ and *Formicoxenus nitidulus*^56^, or the cuckoo bumblebee *B. vestalis*^57^. Although the rise of secondary reproductives in a termite colony differs from social parasites in that they do not kill the previous reproductives, the over-signaling by neotenics could be indicative of the additional functions of secondary-specific compounds, which merit further investigation.

These neotenic-specific compounds may play a role in the sex-specific regulation of reproductives observed in *R. flavipes*^34^. Yet, we did not identify any sex-specific compounds, casting doubt that inhibition of sex-specific reproductive development within a colony is based on CHCs produced by the current king or queen. Similar conclusions have been reported in the termite species *Prorhinotermes simplex*, where chemical compounds distinguish between sterile castes and functional reproductives, but not between sexes of reproductives^58^. Sex-specific inhibition could be mediated through other pathways, as sex-specific compounds have been identified from the proteinaceous secretions of reproductive individuals in three termite species, including an introduced population of *R. flavipes*^27^. Similarly, royal signaling can be mediated through highly volatile compounds, such as the sesquiterpene alcohol (*E*)-nerolidol emitted exclusively by the primary queens and present on the eggs of four higher termite species from the subfamily Syntermitinae^35^. In the dampwood termite *Zootermopsis nevadensis,* headbutting behaviors have been linked to the inhibition of reproductive development^59^. Although there was no sex specificity observed in the behaviors of *Z. nevadensis,* these other forms of communication could potentially work in concert with CHCs to regulate complex functions in the colony, such as the differentiation of reproductives.

Heneicosane and the other ‘neotenic’ royal compounds were not found in all of the neotenics investigated in this study, but only in 75% of the females and 50% of the males (values for heneicosane). This finding differs from the results of Funaro et al.^36^ who reported heneicosane in all seven female and male neotenics from two colonies. Most of the neotenics analyzed by Funaro et al. were worker-derived; the authors stated that nymph-derived neotenics possessed similar chemical profiles to worker-derived neotenics. The difference observed with our study may potentially result from geographic variation in the production of heneicosane, as a recent study also failed to detect heneicosane in worker-derived neotenics in Kentucky, USA^40^. The absence of heneicosane production in some neotenics may suggest that this compound is not solely a caste signal, as one would expect its production by both worker- and nymph-derived neotenics. Potentially, this compound may represent a fertility signal, whereby its intensity increases with the degree of fertility of the individual. In our study, we did not investigate whether the queens that did not produce heneicosane were freshly differentiated into neotenic reproducers, were unmated or had not yet laid eggs. Interestingly, heneicosane was absent from newly differentiated worker-derived neotenics (3–25 days post differentiation) from the Kentucky, USA population^40^. In many social hymenopteran species, queen pheromones consistently increase or decrease with fertility and ovarian activity^60^ or with the hormonal changes underlying reproductive activity^19,20,61^. Some workers of the wasp *Dolichovespula saxonica* may lay eggs even in the presence of the queen^62,63^ and these reproductive workers have CHC profiles intermediate between the queen and the non-reproductive workers^24^. Some compounds are therefore reproductive-specific and fertility-linked, and both reproductive workers and queens shared the same compounds, but in different quantities. Our results revealed that the neotenics of *R. flavipes* do not have CHC profiles intermediate between non-reproducing workers and primary reproductives (Fig. 5). Rather, they produce ‘neotenic’ compounds that are absent from primary reproductives, and the two long-chain compounds that are shared (13- and 11-methylpentatriacontane and 13- and 11-methylheptatriacontane) are produced in higher quantities by the neotenics. Notably, the concentrations of these two compounds did not vary with the concentration of heneicosane, suggesting they may have distinct functions. In primary reproductives, these compounds increased with the age of the individuals, and therefore indirectly with colony size. It is possible that these compounds may simply vary with the age of the colony or seasonality^64–66^. These compounds may also signal the fertility of the individuals, such as that observed in ant colonies^61^. However, the higher production of these compounds by neotenics may cast doubt on the fertility-linked signal of these compounds or their honest use by the neotenics, as their egg laying rate does not exceed that of the primary reproducers, despite the fact that in some cases, the combined reproductive output of the neotenics does^67^.

Future studies may provide further insights into the physiological or behavioral changes inducing the production of the neotenic blends (including heneicosane), as a fraction of the neotenics analyzed did not produce the compounds. For example, future work could test whether the lack of production by some neotenics may be explained by their nymph-derived or worker-derived origin. Future work may also disentangle the function of the diverse compounds present in the CHC profiles of *R. flavipes* soldiers, including some of the reproductive-associated compounds. Similarly, the production of heneicosane or other reproductive-associated compounds could be examined in the introduced range of *R. flavipes*^68^, where colonies are mostly headed by neotenics^69^. Whether the difference in colony structure is associated with different production or ratios of the reproductive compounds may provide insights into the roles and functions of the distinct components. Furthermore, testing the behavioral response of workers and neotenics toward the royal compounds may provide further insights into how information is conveyed and their behavioral functions. In evaluating behavioral or physiological responses to chemical blends, it is recommended to test responses toward individual components as well as various combinations of them to discriminate between redundant components or to detect additive or synergistic effects^70^. Finally, this study also questions whether additional signals, such as proteinaceous secretions or highly volatile compounds may contribute to chemical differentiation between royal individuals in *R. flavipes*. Overall, this study lays important groundwork for determining the potential functions of royal pheromones in a termite species, and calls attention to the possibility that primary reproductives and neotenics are distinct royal entities likely facing different pressures. Determining whether primary reproductives and neotenics share a common goal or face separate selective pressures may contribute to a better understanding of the evolution of royal pheromones and social regulation in termites.

## Materials and methods

A total of 22 colonies of *Reticulitermes flavipes* were collected in Central Texas in spring and summer 2018 (Supplementary information Table S1). Termite colonies were brought back to the lab, removed from their wooden logs and transferred into 10 cm Petri dishes. One worker per colony was sequenced at the mitochondrial 16S gene to confirm species identification, following DNA extraction and sequencing procedures from Aguero et al.^71^ Only one primary king and two mature (i.e.*,* fully physogastric) primary queens were found during the sampling. All the orphaned colonies were kept in the lab and regularly checked for the emergence of secondary reproductives. Fifteen of them produced some neotenics. Only the neotenics clearly differentiated (i.e.*,* physogastric female neotenics and highly pigmented male neotenics) were sampled to avoid any misidentification with a large worker. We did not record whether the secondary reproductives were worker-derived (apterous neotenics) or nymph-derived (brachypterous neotenics), nor whether they were reproductively active. Overall, 15 male neotenics and 61 female neotenics, as well as 35 workers, 10 soldiers and 11 nymphs were collected (See detailed sampling in Supplementary information Table S1). The CHC profiles, including the heneicosane component, were obtained from all these samples.

### Caste-associated heneicosane production in R. flavipes

Alates were collected from five different colonies of *R. flavipes* (Supplementary information S1)*,* sexed (males have visible eighth and ninth tergites, while females have an enlarged seventh tergite which hides the eighth and ninth tergites) and randomly paired in 5-cm petri dishes with damp sawdust, mulch, and wood pieces. As mortality during the first stages of foundation is high, we setup ~ 1000 pairs. CHCs were extracted from male and female alates before pairing, as well as at different periods after mating (2 days, 1 and 2 weeks), after egg production (4 and 6 weeks after pairing), and after the first workers hatched (from 8 weeks after pairing to 1.5 years old). CHC profile changes and heneicosane production was compared between the alates’ colony of origin and across time. Overall, the CHC profiles were obtained for 33 alates, 116 primary queens and 110 primary kings (including one mature primary king and two mature primary queens collected from the field).

### GC–MS and statistical analysis

All termite non-volatile CHCs were extracted following the procedure described in Funaro et al.^36^ Individual termites were freeze-killed for 15 min at − 20 °C and extracted in 200 µL hexane for two minutes with gentle mixing. Extracts were evaporated under a stream of high-purity nitrogen, resuspended in 50 μL of hexane, and transferred to a 100 μL glass conical insert in a 1.5 mL autosampler vial. Samples were then analyzed using a 7890B Agilent Gas chromatograph (GC) and 5977B Agilent Mass Spectrometer (MS). A sample volume of 2 μL was injected in splitless mode using a 7693 Agilent autosampler into a HP-5MS UI column (30 m × 0.250 mm internal diameter × 0.25 μm film thickness; Agilent) with ultrahigh-purity helium as the carrier gas (0.75 mL/min constant flow rate). The GC oven was programmed from 50 to 310 °C at 15 °C/min after an initial delay of 2 min and held at 310 °C for 10 min.

CHC identifications were based on their electron ionization mass spectra, and Kovats indices on the HP-5 column. We only used peaks occurring in at least 10 samples (= 48 compounds). Compound identifications were compared with CHC data from previous studies on *R. flavipes*^37,40,72,73^. Overall, 26 peaks were identified and 22 remained unknown. The relative abundance of each chemical compound was compared among castes and between sexes using the posthoc Dunn test following Kruskal–Wallis test using the *R* package *PMCMR*^74^. The P-values were adjusted for multiple comparisons between each pair of castes using a Bonferoni correction. A Principal Component Analysis was performed to segregate individuals based on their caste and sex according to their CHC profiles using the *R* package *factoextra*^75^. All statistical analyses have been performed on R v.3.6.2^76^.

## Supplementary Information

**Supplementary Information 1**.

## Data Availability

The data reported in this study have been deposited in the Open Science Framework database, https://osf.io (https://doi.org/10.17605/OSF.IO/7J54S).
