# Supplementary material for: Distinct chemical blends produced by different reproductive castes in the subterranean termite Reticulitermes flavipes
Source: Sci Rep. 2021 Feb 24;11:4471. doi: 10.1038/s41598-021-83976-6 (PMC7904765; doi:10.1038/s41598-021-83976-6)
Supplement: Supplementary file 1 — Supplementary Information 1. [file 41598_2021_83976_MOESM1_ESM.pdf]

Supplementary Information

Distinct chemical blends produced by different reproductive castes in the subterranean termite *Reticulitermes flavipes*

Pierre-André Eyer*, Jared Salin, Anjel M. Helms, Edward L. Vargo

Department of Entomology, 2143 TAMU, Texas A&M University, College Station, Texas, 77843-2143, USA

*Correspondence

Pierre-André Eyer

Department of Entomology,

Texas A&M University,

College Station, 77843, Texas, USA

e-mail: pieyer@live.fr

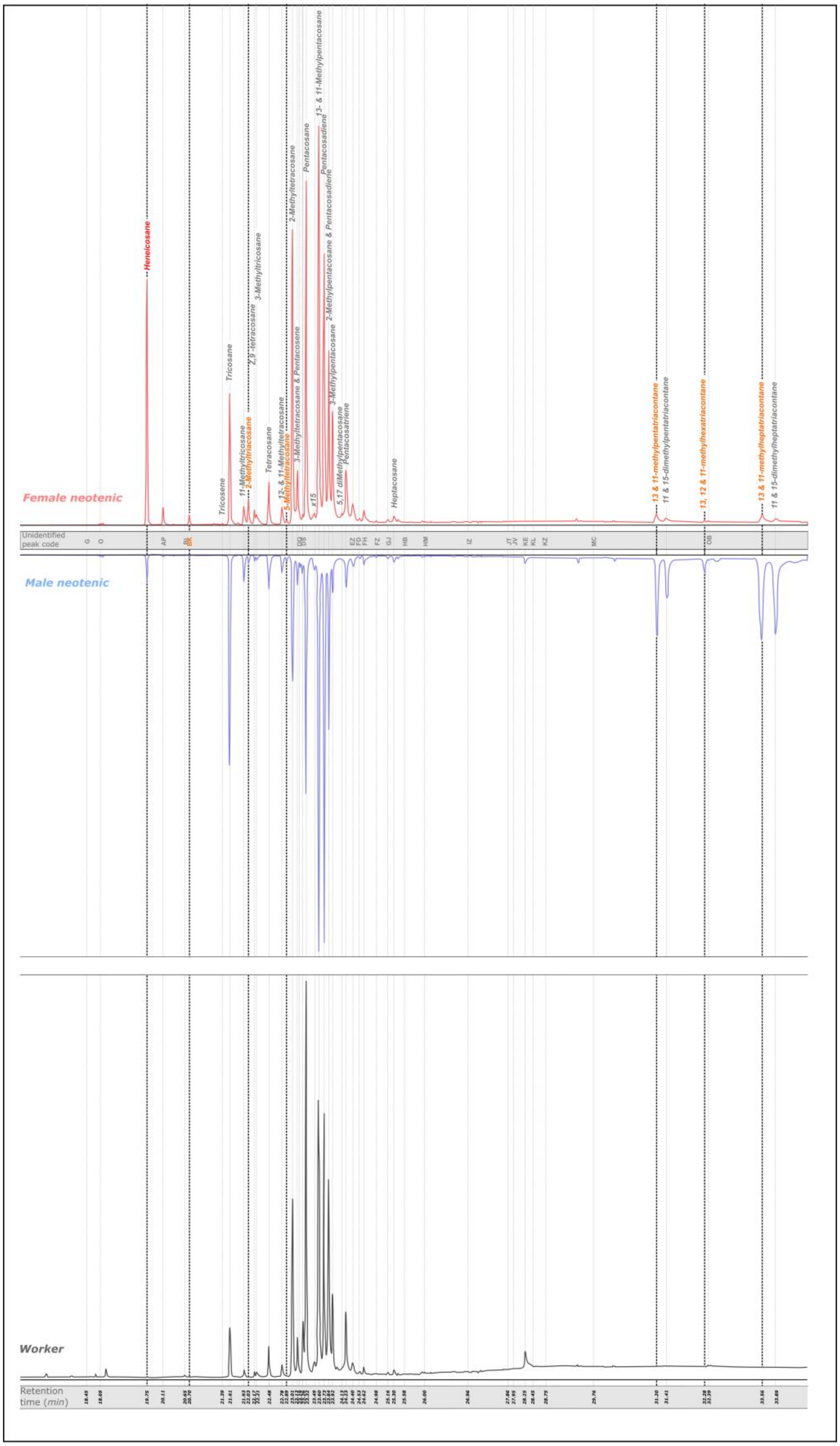

Figure S1a: Gas chromatograms of female and male neotenic, as well as worker in *Reticulitermes flavipes*. Female neotenic profile is marked in red, male neotenic in blue and worker in black.

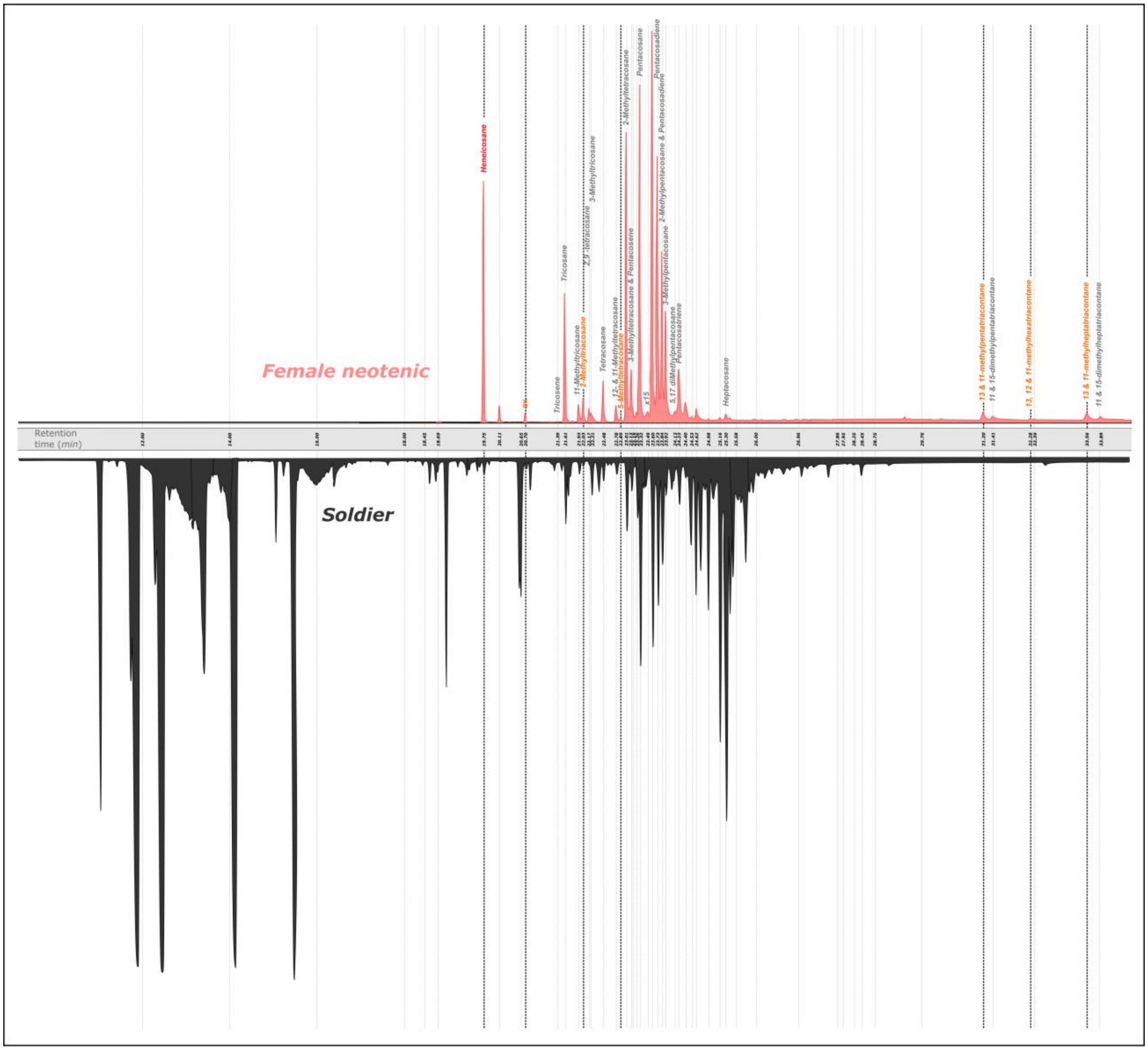

Figure S1b: Gas chromatograms of a female neotenic and a soldier of *Reticulitermes flavipes*.

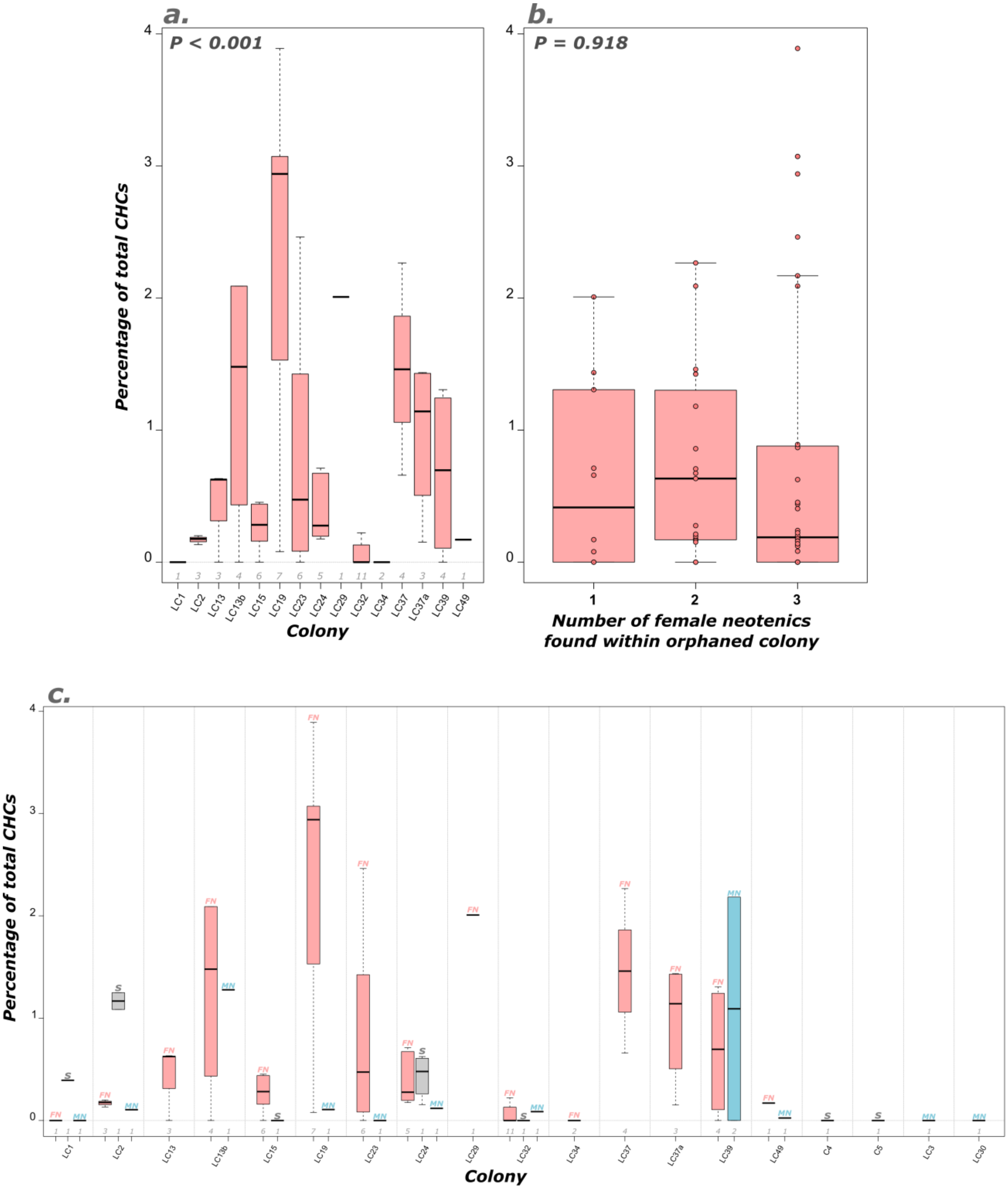

Figure S2: Representation of the relative proportion of heneicosane across female neotenics in function of their colony of origin (***a***) or in function of the number of female neotenics found within orphaned colony (***b***). Representation of the relative proportion of heneicosane across female neotenics (*FN*), male neotenics (*MN*) and soldier (*S*) for each colony (***c***). Small gray numbers indicate sample sizes. Box plots represent median and 1st and 3rd quartile; whiskers include 95% of all observations; individual dots show outlier values.

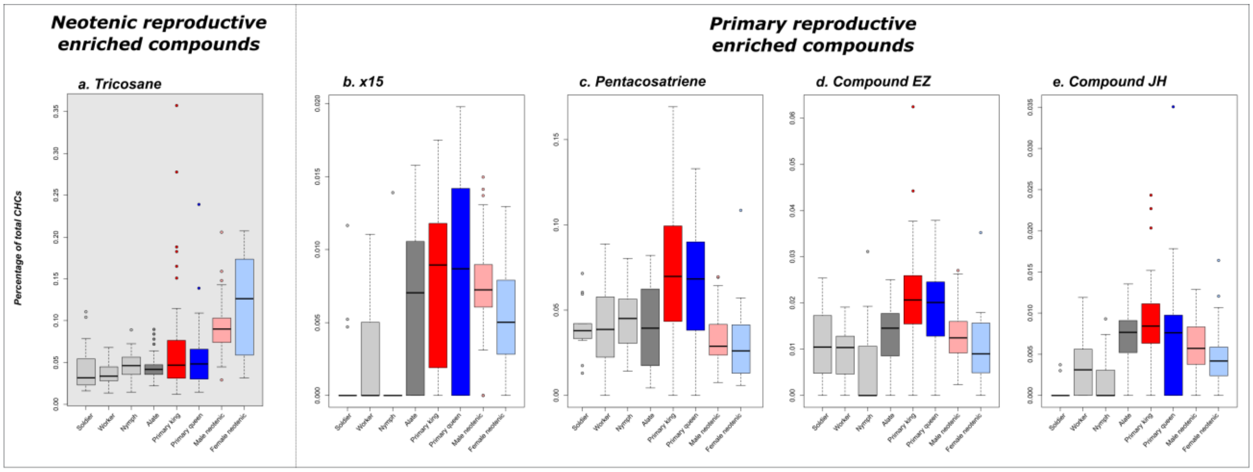

Figure S3: Neotenic- and primary- enriched compounds in *Reticulitermes flavipes*. For each compound, its relative percentage in the total CHCs is represented for every caste sampled.

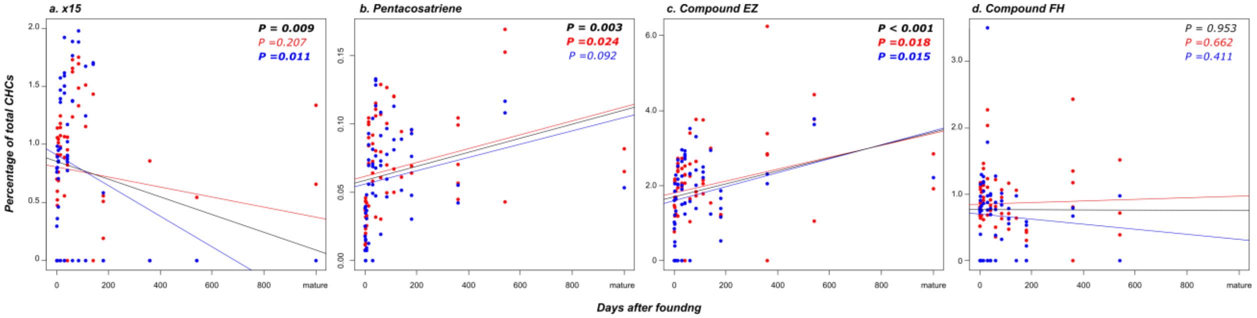

Figure S4: Relationship between the time elapsed since the foundation of incipient colonies and the production of pentacosatriene and the compounds x15, EZ and FH in founding primary reproductives.

Table S1: Sampling information for every individual used in this study.

Colony	Worker	Soldier	Nymph	Alate	Primary		Secondary	
					Queen	King	Queen	king
Orphaned_LC1	2	1					1	1
Orphaned_LC29	4	1					1	1
Orphaned_LC49	2						1	1
Orphaned_LC34							2	
Orphaned_LC2	2	1					3	1
Orphaned_LC39							4	2
Orphaned_LC24	10	3	5				5	1
Orphaned_LC15	2	3	1				6	
Orphaned_LC23	2		2				6	1
Orphaned_LC13			1				7	1
Orphaned_LC19							7	1
Orphaned_LC37							7	
Orphaned_LC32	3		1				11	1
Orphaned_LC3								2
Orphaned_LC30								2
Pairing_CA					4P	8P		
Pairing_C6				8	24P	25P		
Pairing_C3	2			6	22P	23P		
Pairing_C2	4			9	1P	3P		
Pairing_C4	3	1	1	10				
Field_LB1					1M			
Field_LB5	1				1M	1M		
**Total**	37	10	11	33	58	55	61	15

P= Paired in incipient colonies
M = Mature
